# Chemotherapy-induced *miR-141*/MAP4K4 signaling suppresses progression of colorectal cancer

**DOI:** 10.1042/BSR20180978

**Published:** 2018-12-21

**Authors:** Feifei Wang, Lianmei Zhao, Juan Zhang, Zesong Meng, Chaoxi Zhou, Guanglin Wang, Youqiang Liu, Meng Li, Jinchuan Xi, Wenbo Niu, Guiying Wang

**Affiliations:** 1 Department of the Second General Surgery, The Fourth Hospital, Hebei Medical University, Shijiazhuang, Hebei 050011, P.R. China; 2 Research Center, The Fourth Hospital of Hebei Medical University, Shijiazhuang, Hebei 050011, P.R. China; 3 Pediatric Surgery, The Second Hospital of Hebei Medical University, Shijiazhuang, Hebei 050011, P.R. China

**Keywords:** Colorectal cancer, miR-141, MAP4K4, 5-FU

## Abstract

One of the treatment failures for colorectal cancer (CRC) is resistance to chemotherapy drugs. miRNAs have been demonstrated to be a new regulator of pathobiological processes in various tumors. While few studies have explored the specific role of *miR-141* in mediating 5-fluorouracil (5-FU) sensitivity of CRC cells, the present study aimed to detect the contribution of *miR-141* in 5-FU sensitivity. The CRC cells viability was measured by MTS assay and cell colony forming. The expression of *miR-141* and its downstream targets were assessed by reverse transcription quantitative PCR, Western blotting, and immunohistochemistry. The functional assays were conducted using CRC cells and nude mice. At the present study, we found overexpression of *miR-141* could inhibit proliferation, migration, tumor-forming and invasive potential of CRC cells *in vitro* and mitogen-activated protein kinase kinase kinase kinase 4 (MAP4K4) was verified as a directed target of *miR-141*. The combination treatment of *miR-141* with 5-FU, directly targetting MAP4K4, could better inhibit invasion and metastasis of CRC cells colony than either one alone. Furthermore, overexpression of *miR-141*, targetting MAP4K4, enhanced the effected of 5-FU and suppressed the malignant biological behaviors, *in vivo*. Our findings showed that 5-FU inhibited malignant behavior of human CRC cells *in vitro* and *in vivo* by enhancing the efficiency of *miR-141*. Our data suggested that targetting the *miR-141*/MAP4K4 signaling pathway could be a potential molecular target that may enhance chemotherapeutic efficacy in the treatment of CRC.

## Introduction

In China, colorectal cancer (CRC) is the third and fourth most common high mortality cancer of males and females respectively [1]. Surgery including chemotherapy and/or radiotherapy is the most basic treatment method in the stage patients. Approximately 25–50% of patients with metastasis CRC miss the optimal treatment opportunity [2]. Therefore, the effective diagnosis and treatment for CRC are urgent needed. Recently, identifying the molecular target in early diagnosis, therapy and prognosis of cancer have become the focus of anticancer research field. However, the formation mechanism, prevention, and treatment modality of CRC has not been fully illuminated. Therefore, understanding the pathological mechanism of CRC and searching for novel targets for preventing CRC remains to be needed.

miRNAs are a class of noncoding RNA, which play an important role in regulating gene-expression programs, by binding to target genes [3]. Some miRNAs act as tumor suppressors, whereas others, when deregulated overexpressed, can result in the tumor initiation [4,5]. Our previous research has concluded the expression of *miR-141* in the CRC tissues is lower than that of the normal colonic mucosa [6]. Moreover, the up-regulation of mitogen-activated protein kinase kinase kinase kinase 4 (MAP4K4) may be associated with the down-regulation of *miR-141* in the CRC [6]. MAP4K4 belongs to the sterile-20 protein kinase family and is involved in many cellular processes, including cell transformation, adhesion, and motility [7–10]. To explore the functional mechanism of *miR-141* in the CRC, we have performed further research. Meanwhile, we hypothesized *miR-141* affected the role of 5-fluorouracil (5-FU) in inhibiting the progression of CRC. Moreover, we supposed that the chemotherapeutic drugs could play their suppressive impact on CRC cell via disturbing the *miR-141*/ MAP4K4 signal pathway.

## Materials and methods

### Ethics statement

The present experimental methods were performed in accordance with the approved guidelines, which was approved by the Research Center, the Fourth Hospital of Hebei Medical University, Shijiazhuang, China.

### Chemicals

RPMI-1640 medium, FBS and PBS were obtained from Gibco-BRL (Life Technologies, Paisley, Scotland). 5-FU (Sigma Chemical Co., Poole, U.K.) was diluted to 0, 12.5, and 25.0 µg/ml with RPMI-1640 medium. The nucleotide sequences (mimics, mimics control, inhibitor and inhibitor control) of *miRNA-141* were designed and synthesized by RIBOBIO (Shanghai, China). 3-(4,5-dimethylthiazol-2-yl)-5-(3-carboxymethoxyphenyl)-2-(4-sulfophenyl)-2H-tetrazolium, inner salt (MTS) were obtained from Promega Corporation(Madison, WI, U.S.A.).

### MTS and transfections

Human CRC cells of HCT-116 and HCT-8 are kind gift from Dr. Shi Juan (Chinese Academy of Medical Sciences and Peking Union Medical College, Beijing, China). Cell viability was detected by MTS. Cells were seeded in a 96-well plate at a density of 10,000 cells/well for MTS assays cultured RPMI-1640 medium with 10% FBS at 37°C in a 5% CO_2_ cell culture incubator. Twenty microliter of MTS solution was added into each well and incubated for 0, 0.5, 1, 2, and 3 days. Then the optical density absorbance at 492 nm was measured using the microplate reader. HCT-116 and HCT-8 cells were cultured in a six-well plate at 40–60% confluence the day before transfection. *miR-141* was transiently transfected to CRC cells by Lipofectmine 2000 reagent (Invitrogen) based on the manufacturer’s instructions.

### Reverse transcription quantitative PCR and Western blot analysis

To detect the expression levels of *miRNA-141* and MAP4K4 in CRC cells, total RNA was extracted with TRIzol reagent (Invitrogen; Thermo Fisher Scientific, Inc., Waltham, MA, U.S.A.). For the detection of MAP4K4, the primer sequences were listed as follows: MAP4K4 forward, 5′-AAG GAG AGA GCG GGA AGC TA-3′, and reverse, 5′-TTG TTG CAA CTG CCT CTG GA-3′. Glyceraldehyde-3-phosphate dehydrogenase (GAPDH) was used for the internal reference and the primer sequences were listed as follows: GAPDH forward, 5′-GTT GGA GGT CGG AGT CAA CGGA-3′, and reverse, 5′-GAG GGA TCT CGC TCC TGG AGGA-3′. The PCR condition consisted of denaturation at 94°C for 5 min, followed by 94°C for 30 s, 60°C for 30 s, and 72°C for 45 s, for a total of 35 cycles. For the detection of *miRNA-141*, the primer sequences were shown as follows: *miR-141*, 5′-CCG GTA ACA CTG TCT GGT AA-3′. U6 was utilized for the internal reference and the primer sequences were shown as follows: U6, 5′-GCT TCG GCA GCA CAT ATA CTA AAAT-3′. The PCR condition was as follows: denaturation at 95°C for 5 min, followed by 95°C for 15 s, 58°C for 30 s, and 72°C for 30 s, for 40 cycles. The relative quantitation was calculated using the 2^−ΔΔ*C*^_T_ method.

Colon cancer cells were lysed on ice with lysis RIPA buffer (Beyotime Institute of Biotechnology, Haimen, China), containing protease inhibitor. Protein concentration was determined using the BCA kit (Beyotime Institute of Biotechnology). Protein sample (50 ug) was subjected to 12% SDS/PAGE and transferred to a PVDF (Amersham Biosciences, Chicago, IL, U.S.A.). The blot was incubated with 5% skim milk at room temperature for 1 h and then incubated with primary antibodies: MAP4K4(1:1,000; cat. no. ab155583; Abcam, Cambridge, MA, U.S.A.), GAPDH (1:5,000; cat. no. ab9485; Abcam) at 4°C overnight. After washing with Tris Buffered Saline containing Tween (TBST) for three times, the membranes were incubated with goat antirabbit immunoglobulin G (1:3,000; cat. no. ab6721; Abcam) at room temperature for 1 h. Membranes were imaged using the Odyssey imaging system (U.S.A., LI-COR).

### Colony-forming assay

Two hundred cells/well were seeded in a six-well plate and treated with compounds and cultured for 2 weeks in medium containing 10% FBS. After removing the medium, cells were washed with PBS for three times and then fixed with pure methanol for 15 min, and last stained in crystal violet for 25 min. Colony-forming unit of more than 50 cells was counted using the inverted microscope.

### Cell migration and invasion assay

Cell invasion assay was evaluated using Transwell inserts (Corning Costar) with matrigel-coated membrane matrix (8 µm pore sizes). The HCT-116 and HCT-8 cells were treated with *miR-141* mimics, 5-FU, or combination of *miR-141* with 5-FU and then seeded in the upper chamber in the 24-well plate. The medium with FBS was added to the lower chamber. Serum-free medium was added in the lower chamber. The cells were cultured in the incubator at 37°C with 5% CO_2_ for 24 h and then removed with a cotton swab. After being fixed, stained, washed, and air dried, invading cells were counted under a microscope.

Wound-healing assay was conducted to evaluate the migration ability of colon cells. After treated by compounds, colon cells were cultured in a six-well plate at a density of 10^5^ cells each well. At last, the cell monolayer was scratched with a 10-µl pipette tip. Images of the colon cancer cells with different treatment were taken using a microscope every 24 h. The cell-healing rate was calculated.

### Target prediction of *miR-141* and dual-luciferase reporter assay

The target of *miR-141* was analyzed by bioinformatics as previous description. The mRNA 3′-UTR of MAP4K4 containing the predicted binding region or mutated binding region was subcloned into a basicluciferase reporter vector (Promega Corporation, WI, U.S.A.). Vector containing the wild type (WT) *miR-141*-MAP4K4 response element (MAP4K4-WT) and the corresponding mutant (MAP4K4-MUT) were purchase from RiboBio Co Ltd.(Guangzhou, China). The vectors containing WT or mimics controls were cotransfected into HCT-116 cells using Lipofectamine 2000. After 48 h, luciferase activities were detected by the Dual-Luciferase Reporter Assay System (Promega Corporation) according to the manufacturer’s protocol.

### Animal experiment

All above animal experimental procedures were conducted in accordance with National Institutes protocols Health Guide for Care and Use of Laboratory Animals and Ethics Committee of the Fourth Hospital of Hebei Medical University. To conduct xenograft tumor experiment, HCT-116 cells were transfected with lenti-*miR-41* and lenti-NC. After 24 h, cells were harvested and diluted in PBS, and then injected subcutaneously (2 × 10^6^ cells/100 ml/mouse) [11] into the right limb of nude mice (Beijing Vital River Laboratory Animal Technology Co., Ltd). On day 7, the tumor volume was measured using a vernier caliper every 3 days. Once palpable tumors had developed (the 14 days), experimental group mice were treated with 5-FU, 5 mg/kg, every 3 days. The tumor volume was calculated using the following formulate: volume (mm^3^) = 1/2 × length × (width)^2^ [12] The nude mice were sacrificed and the tumors were dissected after 29 days.

### Immunohistochemistry

Immunohistochemistry was performed to detect the expression levels of Ki-67, MAP4K4, and MMP9 in tumor tissue, whose processes are accordance with previous study [13]. The slides of tumor tissue were deparaffinized with xylene and rehydrated through a series of ethanol concentrations. Antigens were retrieved by boiling under pressure in EDTA buffer (pH = 9.0) for 3 min. Sections were incubated with 0.3% H_2_O_2_ for 20 min and blocked with goat serum for 45 min followed by washing with PBS. Sections were then incubated with primary antibodies of Ki-67 (1:100; cat. no. 27309-1-AP; Proteintech, U.S.A.), MAP4K4 (1:100), and MMP9 (1:100; cat no. ab38898; Abcam) at 4°C overnight. The next day, sections were incubated with the secondary antibody at 37°C for 30 min which followed by incubating with the HRP labeled streptavidin solution for 30 min. PBS was used for washing after each step. After being visualized by incubating with 3, 3-diaminobenzidine-tetrachloride (DAB) for 5 min, sections were counterstained with hematoxylin and evaluated by light microscopy.

### Statistical analysis

Data were analyzed by the two-tailed Student’s *t* test using SPSS software (version 18.0; SPSS, Inc., Chicago, IL, U.S.A.) and represented as the mean ± S.D. Statistical significance was ascribed to *P*<0.05.

## Results

### The overexpression of *miR-141* decreased cell proliferation and colony formation in CRC cells

**Figure 1:**
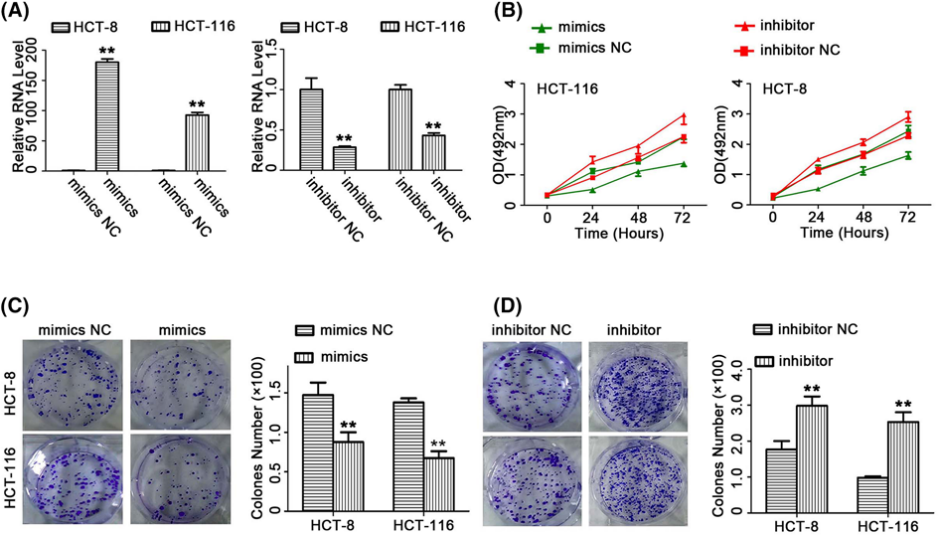
The overexpression of *miR-141* decreased cell proliferation and colony formation in CRC cells HCT-116 and HCT-8 cells were transfected with *miR-141* mimics, *miR-141* inhibitor and control (NC). (**A** and **B**) 24 h after the transfection, cells were harvested for qRT-PCR analysis. The effect of *miR-141* on the viability of CRC cells. Cell viability was determined by MTS assays. (**C** and **D**) representative images from colony formation assay of HCT-116 and HCT-8 cells after transfection of miRNA interference; *n*=3, **P<0.01.

To elucidate the potential role of *miR-141* in CRC cells, we respectively used control, *miR-141* mimics, and *miR-141* inhibitor to transfect CRC cells (HCT-116; HCT-8). Transfection efficiency and expression level of *miR-141* were first evaluated by reverse transcription quantitative PCR (RT-qPCR). The results indicated that the expression of *miR-141* is higher in cells transfected with *miR-141* mimics than that of control, while the expression of *miR-141* is lower in cells transfected with *miR-141* inhibitor than that of control (Figure 1A). We evaluated the effect of *miR-141* over expression and low expression on the proliferation of CRC cells. The low expression of *miR-141* promoted the proliferation of CRC cells (Figure 1B). Up-regulation of *miR-141* inhibited the proliferation of CRC cells. It is reported that *miR-141* is involved in malignant biological behaviors of cancer cells. In the present study, we performed the colony formation assay. Up-regulation of *miR-141* reduced the number of colonies in CRC cells. Depletions of *miR-141* promoted the capability of colony formation in CRC cells (Figure 1C,D).

### *miR-141* inhibited the cell migration and invasion of CRC cells

**Figure 2:**
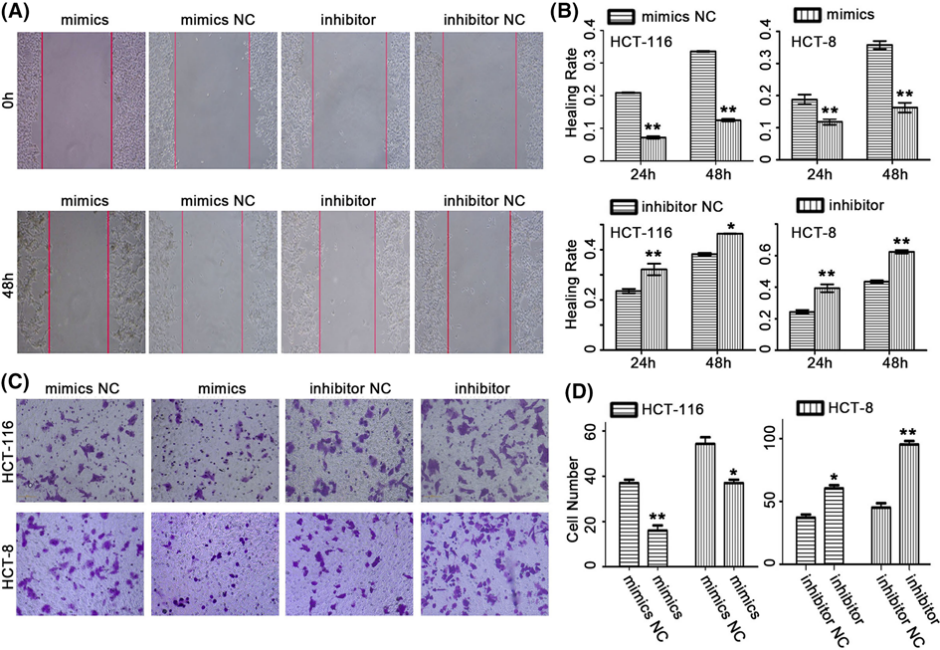
Effects of *miR-141* on CRC cells migration and invasion For the wound-healing and invasion assay, CRC cells were seeded and transfected with *miR-141* mimics, *miR-141* inhibitor and the NC for 24 h. (**A**) Wounds healing from a representative experiment of the HCT-116 cell. (**B**) The wound-healing rate in CRC cells transfected with *miR-141* mimics was significantly decreased, while accelerated in CRC cells transfected with *miR-141* inhibitor compared with the NC. (**C** and **D**) Representative images of the transwell invasion assay. The normal ratio of invasive cells is shown. Up-regulation of *miR-141* in CRC cells reduced cell invasion, while inhibition of *miR-141* expression increased cell invasion; *n*=3, *P<0.05, **P<0.01.

The previous studies have reported *miR-141* is associated with migration. In view of this, we performed the experiments of *miR-141* overexpression and depletion in CRC cells. Next, we conducted the wound-healing assay and transwell experiment. Wound images were captured at 0, 24, and 48 h after scratching. The results demonstrated that overexpression of *miR-141* inhibited the migration of CRC cells, while lowexpression of *miR-141* accelerated the migration of CRC cells (Figure 2A,B). The transwell assay results indicated that overexpression of *miR-141* inhibited the CRC cells invasion. Inversely, lowexpression of *miR-141* accelerated the CRC cells invasion (Figure 2C,D).

### MAP4K4 was a direct target of *miR-141*

To find potential target genes of *miR-141*, we performed the bioinformatics analysis as described before [6]. The result suggested that MAP4K4 could be the important target of *miR-141*. Based on the above result, we further explored the mechanism of *miR-141* in tumor suppression.

**Figure 3:**
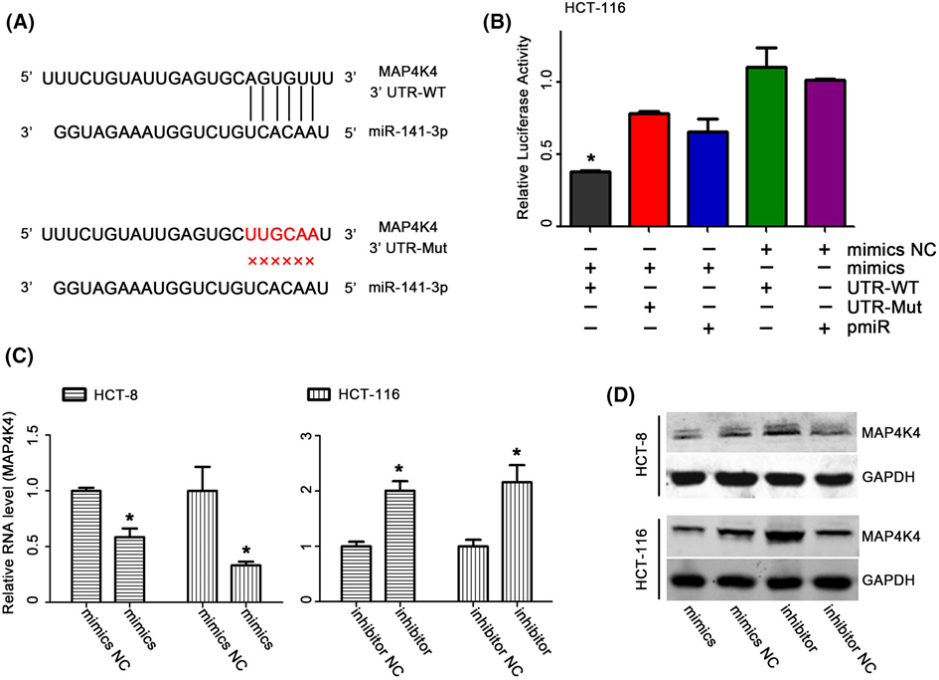
*miR-141* regulated MAP4K4 expression (**A**) predicted *miR-141* target sequence in the 3′-UTR of MAP4K4. (**B**) Dual-luciferasereporter assay of the HCT-116 cells transfected with the MAP4K4-3′-UTR reporter, the MAP4K4-3′-UTR-mutation reporter, *miR-141* mimics, inhibitor, and mimic NC. Being linked to the segment containing the target sequence within the 3′-UTR in MAP4K4 mRNA, *miR-141* expression reduced the luciferase activity. (**C**) 24 h h after the transfection, cells were harvested for RT-qPCR analysis using *miR-141* primers. Overexpression of *miR-141* significantly reduced the MAP4K4 mRNA expression levels in CRC cells. (**D**) MAP4K4 proteins levels in CRC cells treated with *miR-141* mimics, inhibitors and NC; *n*=3, *P<0.05.

To further determine whether *miR-141* suppressed CRC cells by targetting MAP4K4, we conducted MAP4K4-3′-UTR luciferase reporter assays. First, we constructed MAP4K4-3′-UTR-luciferase reporter plasmids [11] for CRC cells transfection. CRC cells were cotransfected with *miR-141* mimics and luciferase reporter plasmid MAP4K4-3′-UTR-luc. In line with the bioinformatics prediction, the transfection of *miR-141* mimics significantly reduced the luciferase activity in CRC cells transfected with MAP4K4-3′-UTR-luc (Figure 3B).

To determine whether *miR-141* could regulate the expression of MAP4K4, we conducted the transient transfection study in CRC cells. Our RT-qPCR assay revealed that the expression of MAP4K4 was significantly decreased (*P*<0.05) in cells transfected with *miR-141* mimics compared with the control. On the contrary, the expression of MAP4K4 was observably induced (*P*<0.05) in cells transfected with *miR-141* inhibitor (Figure 3C).

To further explore the function of *miR-141* in regulating CRC progression, we performed transfection study in CRC cells. Our Western blot assay indicated that overexpression of *miR-141* significantly decreased the protein level of MAP4K4 (Figure 3D). At the same time, down-regulation of *miR-141* significantly increased the protein level of MAP4K4 (Figure 3D).

### 5-FU suppressed CRC cells movement via regulating *miR-141*-mediated MAP4K4 axis

**Figure 4:**
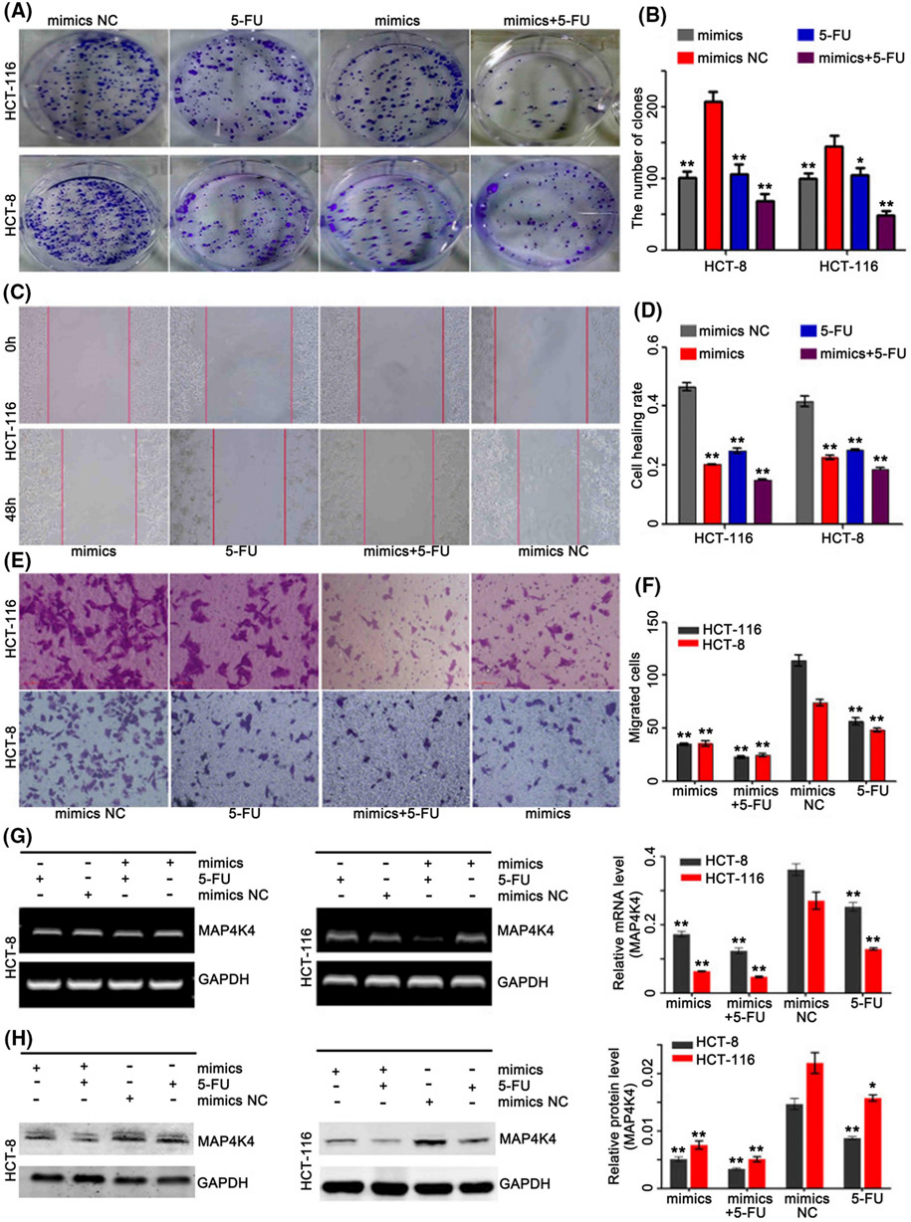
5-FU treatment suppressed CRC cells movement via regulating *miR-141*-mediated MAP4K4 axis (**A** and **B**) representative results of colony formation of 5-FU, *miR-141* mimics, combination of the two and NC. (**C** and **D**) Wound-healing assay was conducted and phase-contrast images were obtained immediately after wounding and at 48 h. (**E** and **F**) Invasion assay was performed. Representative images were recorded. (**G** and **H**) Expression level of MAP4K4 mRNA and proteins were assessed in CRC cells treated by 5-FU, *miR-141* mimics, combination of the two and NC; *n*=3, *P<0.05, **P<0.01.

**Figure 5:**
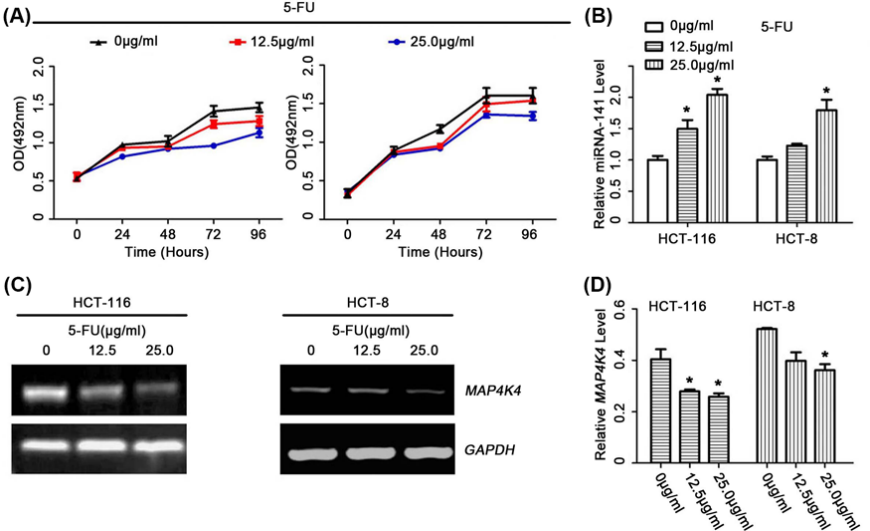
After treatment of CRC cells with 5-FU, levels of MAP4K4 genes following *miR-141* also subsequently changed (**A**) CRC cell viability was detected with MTS assays. (**B**) The expression level of *miR-141* was detected by RT-qPCR assays in the CC cells treated with different concentration of 5-FU. (**C**) The expression level of MAP4K4 genes was detected by PCR in CRC cells treated with different concentration of 5-FU; n = 3, *P<0.05.

Invasion, metastasis, proliferation, and colony forming are the markers of cancer cells. We found that 5-FU inhibited cell colony formation, migration and invasion, and its inhibitory effects overexpressed cells were stronger than the control cells (Figure 4A–F). We also found that the 5-FU could inhibit the proliferation of CRC cells and decrease the expression of MAP4K4, in dose dependent manners (Figure 5).

The present study indicated that *miR-141* could regulate the expression of MAP4K4 in CRC cells. To explore the effective and potential mechanism of *miR-141* in CRC chemoresistance, we performed the Western blot assay and PCR. We found that MAP4K4 was also regulated by *miR-141* in the process of chemosensitivity. The PCR and Western blot assays results showed that combined *miR-141* overexpression and 5-FU significantly decreased the expression of MAP4K4, compared with the control (Figure 4G,H).

### *miR-141* sensitized CRC cells to 5-FU treatment *in vivo*

**Figure 6:**
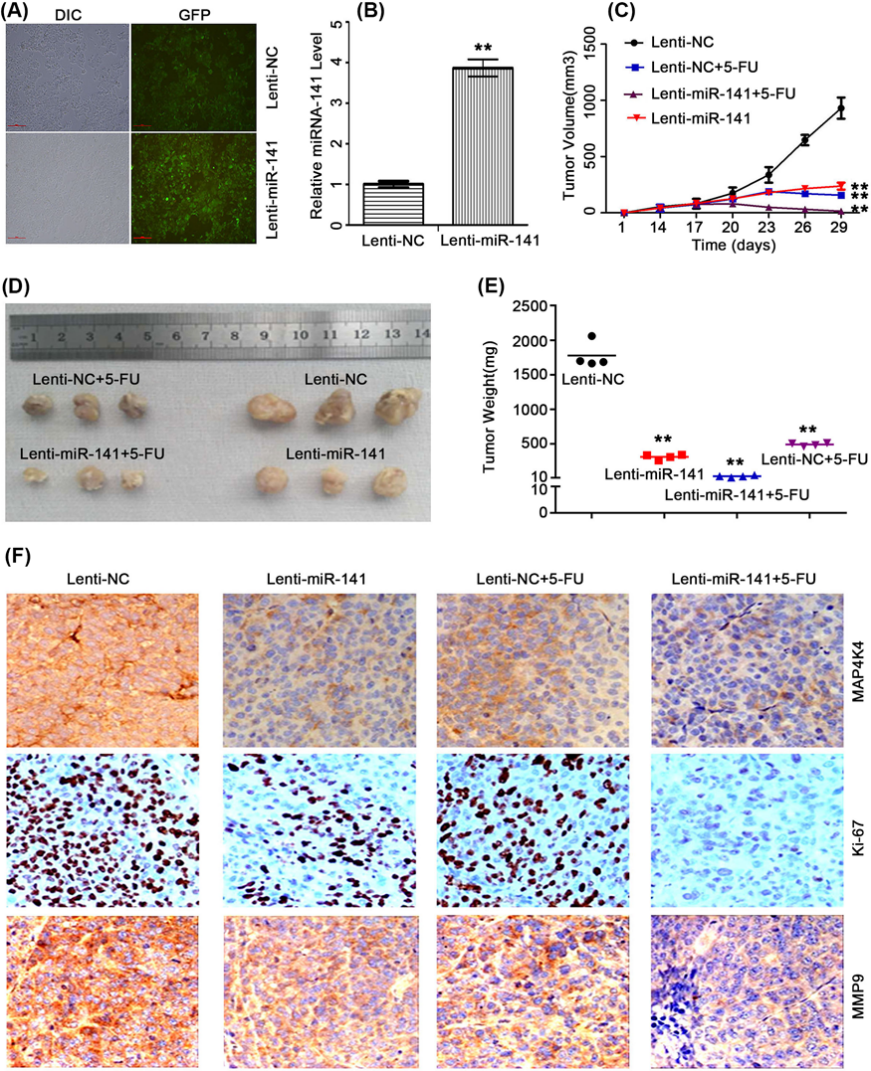
*miR-141* and 5-FU could reverse the colon cancer tumorigenesis Each representative images treatment group are presented the effect of 5-FU, *miR-141*, and combined the two on colon tumors in the nude mice. (**A**) Phase-contrast and flurescence images of HCT-116 transfected with lenti-*miR-141*. (**B**) RT-qPCR analysis of *miR-141* in HCT-116 transfected with Lenti-*miR-141* or Lenti-NC. (**C, D**, and **E**) Tumor weight and volume of lenti-NC+5-FU, lenti-NC, lenti-miR-141 + 5FU, and lenti-*miR-141* cells were recorded. (**F**) The expression levels of MAP4K4, Ki-67, and MMP9 in the tumors tissues of mice in different groups were determined by immunohistochemistry (×200); *n*=3, **P<0.01.

To further confirm the above findings, we performed tumor formation in nude mice to validate the effect of *miR-141* on 5-FU-sensitivity. Lenti-*miR-141* and Lenti-NC cells were respectively injected into the right limb of nude mice. After treatment with 5-FU, tumors derived from *miR-141*-overexpressing cells grew more slowly and had lower tumor weight than the control (Figure 6D,C).

To explore the molecular change in each of the treatment group, we conducted the immunohistochemistry staining. The expression analysis of Ki-67, MMP9 and MAP4K4 indicated that miR-141 can sensitize CRC cells to 5-FU (Figure 6F).

## Discussion

Because of the introduction of the currently used FOLFOX and FOLFIRI regiments in metastatic CRC, the response rates have been improved to about 30–40% [14]; however, the resistance to chemotherapy is still a major barrier in the CRC treatment. Most deaths in CRC are attributed to the resistance to chemotherapeutic drugs. Varied mechanisms are explored to find the novel target. *miR-21* functions as the chemical resistance for cyclo-oxygenase-2 inhibitor, which prevents gastric carcinoma [15]. *miR-141* is highly overexpressed and PTEN is inhibited in 5-FU and oxaliplatin chemo-resistance to esophageal cancer cell [16]. Shota’s study showed that the expression levels of *miR-200c* and *miR-141* were significantly reduced in oxaliplatin-resistant CRC cells [17]. *miR-20b* reduces 5-FU resistance by regulating the expression of ADAM9/EGFR in CRC [18]. In the present study, the major finding was that *miR-141* enhanced the CRC chemosensitivity of 5-FU *in vitro* and tumor xenografts *in vivo* by inhibiting MAP4K4 expression.

miRNAs, 18–25 nts long, are abundant and evolutionary conserved single-stranded RNAs. It is reported that miRNAs account for 1–2% of the human genome and regulate more than 50% protein-coding genes. Compared with the normal counterpart, miRNAs are up- or down-regulated in malignant tissues, which can be considered as oncogenes or tumor-suppressors, respectively [19]. Some studies showed that miRNAs dysregulation in the human is relevant to clinical course cancer. The present study showed that *miR-141* suppressed CRC cell migration, invasion, and proliferation, which indicated that *miR-141* was associated with CRC progression. Depending on the subcellular localization, the function of *miR-141* (tumor suppressor or oncogene) is different. Liu’s research showed that *miR-141* suppressed the proliferation, invasion, and metastasis of the prostate cancer cell by multiple mechanisms [20]. Expression of *miR-141* and MEG3 can abolish the expression of E2F3 and inhibit the process of gastric cancer [21]. Our previous study showed that the expression of *miR-141* was dramatically decreased in tumor tissues and positive lymph nodes [6]. This indicated that *miR-141* was an important tumor suppressor. Deregulation of *miR-141* has been showed in human tumors. *miR-141* could suppress CRC cells proliferation and migration [22]. A previous research indicated that ELF3 promotes epithelial-mesenchymal transition (EMT) by regulating *miR-141-3p* leading to the enhancement of ZEB1, in hepatocellular carcinoma [23]. Here, our results showed that comparison with down-regulated CRC cells, up-regulated *miR-141* expression inhibited tumor progression, *in vivo* and *in vitro*.

We conducted luciferase reporter assays and the result indicated a dramatic reduction in MAP4K4 protein levels in the CRC cells treated with *miR-141* mimics, but down-regulation of *miR-141* had a contrary result. The present study revealed that MAP4K4 was a novel target gene of *miR-141*, and the deregulation of *miR-141*/MAP4K4 contributed to CRC progression. MAP4K4 participate in the maintenance of malignant phenotype of many human cancers. A study showed knockdown of MAP4K4 inhibited progression of lung adenocarcinoma *in vivo* and *in vitro* [24]. EMT and invasion were facilitated by MAP4K4-activating JNK and NF-κB signaling, in hepatocellular carcinoma cells [25]. Our previous data also demonstrated that the MAP4K4 were significantly up-regulated in the tumor and lymph nodes of CRC patients [6]. Recent researches indicate that the Ki-67 protein exists in the cell nucleus during mitosis, regulates the cell cycle. MMP9 promotes tumor metastasis by facilitating tumor cell migration and invasion. Furthermore, our results indicated that the combination of up-regulated *miR-141* with 5-FU could inhibit the expression of Ki-67 and MMP9 proteins. In the present study, cell proliferation, migration, and invasion were inhibited when *miR-141*/MAP4K4 was elevated. In summary, the present data certified a correlation between the *miR-141* and both sensitive to 5-FU and MAP4K4.

In the present study, the *miR-141*-mediated MAP4K4 axis was the novel target of 5-FU in CRC cells. The present study showed that MAP4K4 was the target gene of *miR-141*. Downexpression of MAP4K4 prevented the growth of tumor. MAP4K4 regulates the expression of kinase, transcription factor, transmembrane receptor that is important for cell–cell communication and matrix metalloproteinases in tumor [26,27]. However, the function of MMP9 and Ki-67 in the process of 5-FU anticancer has not been illuminated. And large effectors or signaling mediators are not explored. Our study showed that MAP4K4 play an important role in CRC progression.

The present study showed that the synergistic role of overexpressed *miR-141* and 5-FU lead to the decreased expression of MAP4K4. In conclusion, our results demonstrated loss of *miR-141*/MAP4K4 contributed to the progression of CRC. Up-regulating *miR-141* expression may represent a novel therapeutic method approach and prevention for CRC.

## Abbreviations

5-FU: 5-fluorouracil
CRC: colorectal cancer
EMT: epithelial-mesenchymal transition
E2F3: E2F transcription factor 3
GAPDH: glyceraldehyde-3-phosphate dehydrogenase
Ki-67: proliferating antigen Ki-67
MAP4K4: mitogen-activated protein kinase kinase kinase kinase 4
MMP9: matrix metalloproteinase-9
PTEN: phosphatase and tensin
RT-qPCR: reverse transcription quantitative PCR
WT: wild type
U6: snRNA internal control gene
